# Endometriosis: from iron and macrophages to exosomes. Is the sky clearing?

**DOI:** 10.1093/hropen/hoae034

**Published:** 2024-05-30

**Authors:** Jacques Donnez, Marie-Madeleine Dolmans

**Affiliations:** Department of Gynecology, Université Catholique de Louvain, Brussels, Belgium; Société de Recherche pour l’Infertilité (SRI), Infertility Research Department, Brussels, Belgium; Gynecology Research Department, Institut de Recherche Expérimentale et Clinique, Université Catholique de Louvain, Brussels, Belgium; Department of Gynecology, Cliniques Universitaires Saint-Luc, Brussels, Belgium

Determining the pathogenesis of endometriosis remains a challenge for gynecologists, reproduction specialists, endocrinologists, and researchers alike. Multifactorial causes have been reported, including ectopic endometrial tissue, altered immunity, unbalanced cell proliferation and apoptosis, oxidative stress and reactive oxygen species (ROS), aberrant endocrine signaling, and genetic factors (Bulun *et al.*, 2019; Kapoor *et al.*, 2021; Taylor *et al.*, 2021; Donnez and Cacciottola, 2022; Vercellini *et al.* 2024). However, a very comprehensive understanding is needed to detect and investigate the physiological, cytological and immunological events, as well as biochemical factors like oxidative stress and inflammation, encountered in the pelvic microenvironment of endometriosis patients. In this issue of *Human Reproduction Open*, Chaggar *et al.* (2024) report high rates of deep endometriosis induced by hemoperitoneum. In our invited commentary, we review the role of blood, hemoglobin (Hb), and iron in endometriosis pathogenesis, looking to elucidate the possible link suggested in this article.

## Hemoglobin and iron

**Figure 1.**
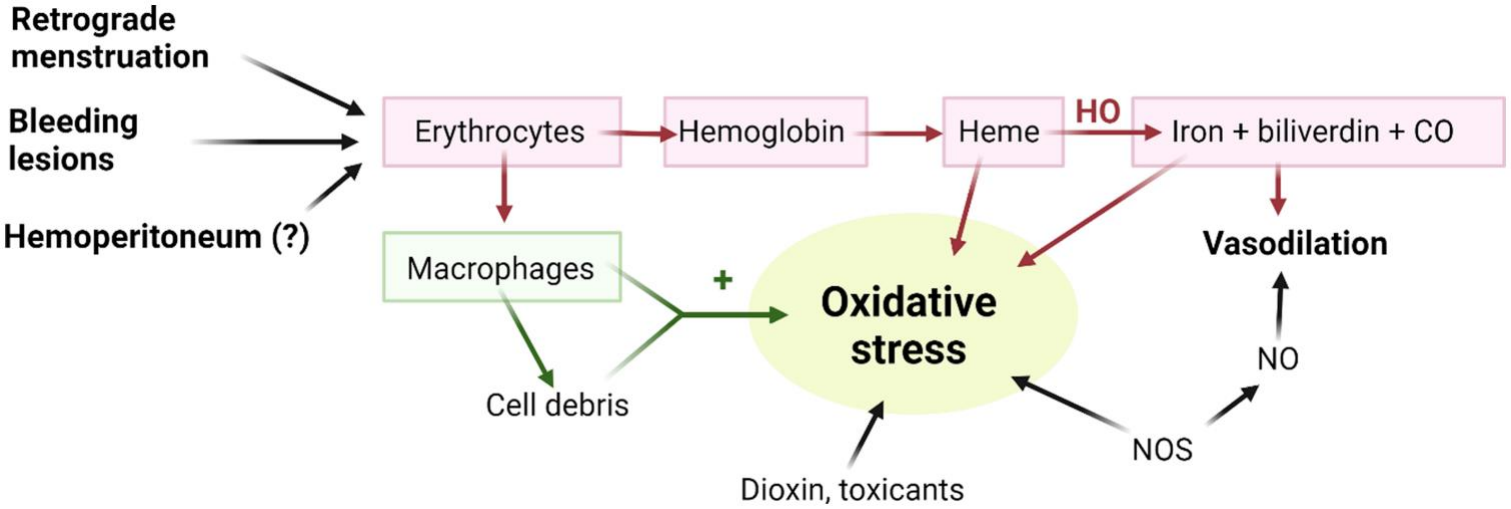
**Erythrocytes carried into the peritoneal cavity by menstrual reflux, bleeding endometrial lesions or hemoperitoneum, hemoglobin and its highly toxic by-products (heme and iron), and macrophages, inducing oxidative stress**. Activated macrophages are also able to deliver various inflammatory molecules and trigger oxidative stress. CO, carbon monoxide; HO, heme oxygenase; NO, nitric oxide; NOS, nitric oxide synthase.

**Figure 2.**
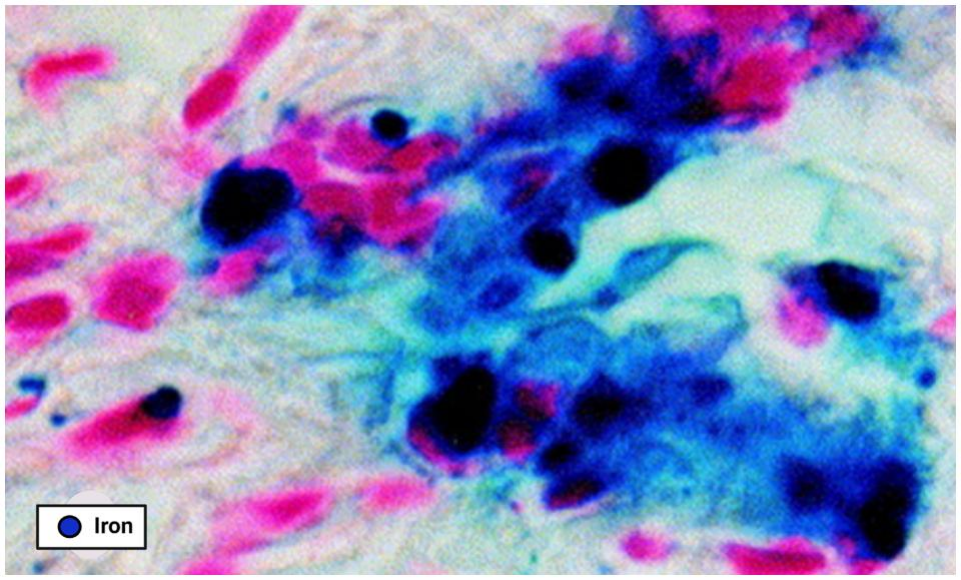
**Iron-overloaded macrophages in an endometriotic lesion identified by Prussian blue staining**. Activated macrophages are highly engaged in erythrocyte degradation, as suggested by the presence of numerous iron-loaded macrophages in peritoneal fluid and lesions from endometriosis patients (from Van Langendonckt *et al.*, 2002b, with permission).

Erythrocytes carried into the peritoneal cavity by menstrual reflux and/or bleeding lesions are known to be inducers of oxidative stress (Van Langendonckt *et al.*, 2002a,b; Defrère *et al.*, 2006, 2008, 2011; Lousse *et al.*, 2009, 2012). Indeed, erythrocytes are likely to release pro-oxidant and proinflammatory factors like Hb and its highly toxic by-products heme and iron into the peritoneal environment (Van Langendonckt *et al.*, 2002a,b) (Fig. 1). Unless they are properly chelated, free iron and heme become key players in the formation of deleterious ROS (Van Langendonckt *et al.*, 2002a,b; Agarwal *et al.*, 2005). Several *in vitro* studies (Defrère *et al.*, 2006; Lousse *et al.*, 2009) have demonstrated the involvement of iron overload in the proliferation of endometriotic lesions induced in murine models. This strongly suggests that iron is implicated in endometriosis development in women, as demonstrated by the presence of iron-loaded macrophages in peritoneal endometriotic lesions in affected individuals (Van Langendonckt *et al.*, 2002a,b) (Fig. 2). Iron conglomerates containing hemosiderin, another form of iron storage found in cases of iron overload, have also been witnessed in endometriotic lesions (Van Langendonckt *et al.*, 2002b). Indeed, erythrocytes reside in the peritoneal cavity of most (90%) menstruating women, so why do some individuals develop endometriotic lesions and others not? One hypothesis states that peritoneal protective mechanisms are swamped by menstrual reflux in some patients, either because of its abundance or due to defective scavenging systems (Donnez *et al.*, 2016; Van Langendonckt *et al.*, 2002a,b). A key defense mechanism to counteract the effects of hemorrhage is mediated by haptoglobin (Hp), which is able to bind to extracellular Hb, thereby attenuating its oxidative and inflammatory potential (Donnez *et al.*, 2016).

For more than 20 years now, we have been claiming that iron plays a crucial role in endometriosis (Van Langendonckt *et al.*, 2002a,b) and advocating use of iron chelators, since they were shown to prevent initiation and progression of the disease in murine models (Defrère *et al.*, 2011). Despite our findings, iron chelators were never developed in clinical research for treatment of endometriosis, but the role of highly toxic Hb by-products like iron was highlighted in two recent reviews published by Wyatt *et al.* (2023) and Vercellini *et al.* (2024).

## Key role of macrophages in iron metabolism

**Figure 3.**
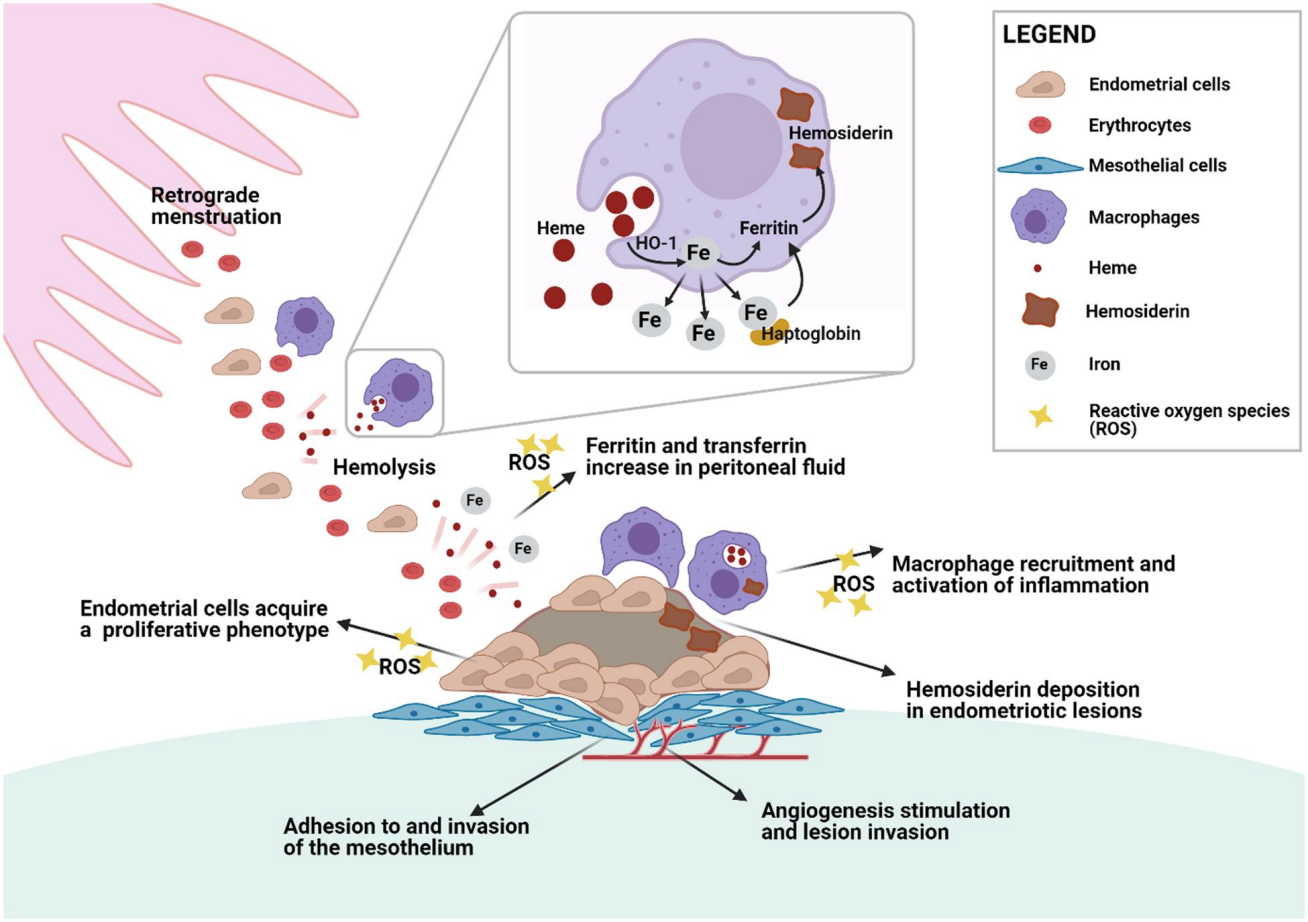
**Endometrial cell and macrophage interaction in the pelvic cavity**. Erythrocytes and endometrial cells are carried into the pelvic cavity by retrograde menstruation and phagocytosed by peritoneal macrophages. Heme digestion by HO-1 releases iron, which is either stored in the form of ferritin and hemosiderin or released to bind to transferrin. Endometrial cells with adhesive characteristics start to invade the mesothelium and trigger inflammatory signals that recruit more peritoneal macrophages. Local inflammation and increased levels of ROS contribute to acquisition of a proliferative phenotype and proangiogenic features crucial to endometriotic lesion development (adapted from Cacciottola *et al.*, 2021). HO-1, heme oxygenase-1; ROS, reactive oxygen species.

Neutrophils, macrophages, natural killer (NK) cells, and dendritic cells are cell populations of the innate immune system predominantly involved in endometriosis pathogenesis (Kapoor *et al.*, 2021; Gajbhiye, 2023). Macrophages are immune cells charged with detecting foreign elements in the system and subsequently destroying them. Iron metabolism and the role of macrophages in the pelvic cavity in endometriosis pathology are graphically postulated in Fig. 3. Activated macrophages recruited inside the pelvic cavity are deeply engaged in degradation of erythrocytes, as indicated by numerous iron-loaded macrophages in the peritoneal fluid from both endometriosis patients and mice intraperitoneally injected with erythrocytes. Macrophages typically phagocytose senescent erythrocytes or endocytose the Hb–Hp complex. Hb and heme degradation by heme oxygenase (HO) release iron, which is then incorporated into ferritin inside macrophages or sent back to the iron transporter transferrin via peritoneal fluid.

A number of studies have emphasized the involvement of peritoneal macrophages in iron metabolism (Van Langendonckt *et al.*, 2002a,b; Taylor *et al.*, 2021). Cellular iron storage within ferritin hampers the ability of iron to generate free radicals and thereby confers an antioxidant effect. However, ongoing delivery of iron to macrophages may overwhelm the capacity of ferritin to store and sequester the metal, causing oxidative injury to cells (Van Langendonckt *et al.*, 2002a,b). We hypothesized in 2016 that the iron detoxification system could be progressively overwhelmed during the menstrual cycle in endometriosis patients, leading to abnormal macrophage activation (Donnez *et al.*, 2016). By releasing cytokines that trigger other cells, activated macrophages initiate the process of inflammation. In this way, iron overload induces oxidative stress.

## Heme oxygenase and detoxification systems

HO-1 is a heme-degrading enzyme strongly upregulated by heme. It protects cells from heme-generated oxidative stress by producing beneficial molecules that deliver unique protective and antioxidant effects, including carbon monoxide, bilirubin, and biliverdin (Van Langendonckt *et al.*, 2002a,b; Donnez *et al.*, 2016). HO-1 induction is also associated with increased ferritin synthesis, free iron scavenging, and ensuing protection against any negative repercussions.

However, in endometriosis, inducible HO-1 shows weak expression by macrophages and mesothelial cells, which make up the majority of cells in the peritoneal cavity, and there is no concomitant upturn in peritoneal fluid levels of bilirubin, its final byproduct. All this strongly suggests that detoxifying systems, while present, might be insufficient to metabolize Hb in the case of endometriosis (Donnez *et al.*, 2016) or peritoneal hemoperitoneum, as in the series reported by Chaggar *et al.* (2024).

## Activated macrophages and inflammatory molecules: their role in disease progression

In the uterine environment, the function of all immune cells, including macrophages, NK cells, and T cells, is regulated by associated increases in levels of proinflammatory mediators (Cacciottola *et al.*, 2021; Kapoor *et al.*, 2021; Taylor *et al.*, 2021; Nazri *et al.*, 2023; Oală *et al.*, 2024). Proinflammatory pathways prevent apoptotic pathways from clearing debris, so these unwanted cells may travel and adhere to distant sites.

Macrophages are able to deliver various inflammatory molecules that are responsible for both initiation and progression of endometriosis (Taylor *et al.*, 2021; Dolmans and Donnez, 2022; Donnez and Cacciottola, 2022; Ni and Li, 2024) (Fig. 3). They are also known for their wide-ranging functional and phenotypic alterations (Nazri *et al.*, 2020; Dolmans and Donnez, 2022). These changes are governed by stimuli like oxidative stress, tissue damage, and hormones, leading to activation of different pathways of proliferation, migration, and invasion (Agarwal *et al.*, 2005; Donnez *et al.*, 2016).

Macrophage migration inhibitory factor is an inflammatory cytokine that assumes a critical function in the early development of endometriosis (Chekini *et al.*, 2021). It recruits macrophages into endometriotic lesions and helps them proliferate by release of proinflammatory cytokines and other growth factors (Cacciottola *et al.*, 2021). Stratopoulou *et al.* (2023) investigated the role of M2 macrophages in endometrial invasiveness in adenomyosis. They found that accumulation of M2 macrophages enhances the invasion capacity of endometrial cells. In their model, M2 macrophage infiltration was sufficient to promote the disease and its progression. They raised the possibility of collective cell migration (CCM) involvement in the invasion process of myometrium by endometrium. CCM was also demonstrated in a baboon model of endometriosis, mimicking the invasion process seen in endometriosis (Donnez *et al.*, 2015; Orellana *et al.*, 2017).

As several papers (Stratopoulou *et al.*, 2021; Donnez *et al.*, 2024) have indeed confirmed common pathogenic features in both deep endometriosis and adenomyosis, namely excessive macrophage accumulation, fibrosis, and irregular angiogenesis, why not go further and extrapolate that infiltration by activated macrophages is pivotal to invasion by endometrial cells in both diseases?

## Reactive oxygen species and oxidative stress

ROS are intermediaries produced by normal oxygen metabolism, but are known to have deleterious effects (Agarwal *et al.*, 2005). To protect themselves, cells have developed a wide range of antioxidant systems to limit ROS production, inactivate the molecules, and repair cell damage. In healthy individuals, ROS and antioxidants are in balance. However, when the balance is tipped toward an overabundance of ROS, oxidative stress ensues and can impact the reproductive lifespan of women (Donnez *et al.*, 2016; Cacciottola *et al.*, 2021). Oxidative stress occurs when the balance between ROS production and antioxidant defense is disrupted due to either inadequate antioxidant protection or excess production of ROS. Various lines of evidence support the role of oxidants in the development of endometriosis, since endometriotic cells show higher endogenous oxidative stress levels, elevated ROS production, and alterations to ROS detoxification pathways (Donnez *et al.*, 2016).

First of all, Hb, heme, and iron derivatives are generated from hemolysis of erythrocytes abnormally accumulating in endometriotic lesions. Second, the ability to survive the oxidative activity of these derivatives appears to be conducive to endometriotic cell growth. Lower levels of apoptosis observed in lesions suggest that aberrant adenomyotic and endometriotic cells may survive and contribute to progression of the disease (d’Argent *et al.*, 2023). Finally, endometriotic lesions residing in their unique microenvironment may display significant individual differences in terms of degree of responsiveness to free radicals or antioxidant defenses (Donnez *et al.*, 2016). Investigating the mechanisms underlying oxidative stress associated with endometriosis may well prove fruitful for determining the specific pathways responsible for initiation and progression of the disease (Kapoor *et al.*, 2021; Dolmans and Donnez, 2022).

## The future

Small extracellular vesicles (sEVs) (<200 nm) are cell-derived vesicles containing microRNAs (miRNAs) that regulate post-transcriptional gene expression. In 2020, Nazri *et al.* (2020) characterized exosomes found in peritoneal fluid from endometriosis patients. In a very recent paper, Zipponi *et al.* (2024) proved the feasibility of *in vitro* culture of the endometrioma wall and managed to isolate and examine secreted exosomes. Analysis of miRNA exosome content and predicted target genes may well prove to be a promising starting point for a better understanding of endometriosis pathogenesis, addressing the potential influence of miRNA expression in sEVs secreted by lesions and macrophages from women with the disease. Characterization of exosomes opens up brand new avenues for diagnosis and investigation of endometriosis (Nazri *et al.*, 2023; Zipponi *et al.*, 2024).

## Conclusion

There is no doubt that the pathogenesis of endometriosis is multifactorial. It is also clear that iron overload, delivery of inflammatory molecules by activated macrophages, and oxidative stress create a favorable environment for endometrial cells to implant, progress, and metastasize to other locations. Iron overload in the pelvic cavity and its consequences (activation of macrophages and oxidative stress) could potentially be the link explaining the high incidence of endometriosis after hemoperitoneum, as reported in the current issue of *Human Reproduction Open* by Chaggar *et al.* (2024).
